# The Role of Histone Variants in the Epithelial-To-Mesenchymal Transition

**DOI:** 10.3390/cells9112499

**Published:** 2020-11-17

**Authors:** Imtiaz Nisar Lone, Burcu Sengez, Ali Hamiche, Stefan Dimitrov, Hani Alotaibi

**Affiliations:** 1 Izmir Biomedicine and Genome Center, Izmir 35340, Turkey; imtiaznisar.lone@ibg.edu.tr (I.N.L.); burcu.sengez@ibg.edu.tr (B.S.); stefan.dimitrov@univ-grenoble-alpes.fr (S.D.); 2 Izmir International Biomedicine and Genome Institute, Dokuz Eylül University, Izmir 35340, Turkey; 3 Institute of Genetics and Molecular and Cellular Biology (IGBMC), 1 rue Laurent Fries, 67400 Illkirch, France; hamiche@igbmc.fr; 4 Université Grenoble Alpes, CNRS UMR 5309, INSERM U1209, Institute for Advanced Biosciences (IAB), Site Santé-Allée des Alpes, 38700 La Tronche, France

**Keywords:** epithelial-to-mesenchymal transition, epigenetics, chromatin, nucleosomes, histone variants

## Abstract

The epithelial-to-mesenchymal transition (EMT) is a physiological process activated during early embryogenesis, which continues to shape tissues and organs later on. It is also hijacked by tumor cells during metastasis. The regulation of EMT has been the focus of many research groups culminating in the last few years and resulting in an elaborate transcriptional network buildup. However, the implication of epigenetic factors in the control of EMT is still in its infancy. Recent discoveries pointed out that histone variants, which are key epigenetic players, appear to be involved in EMT control. This review summarizes the available data on histone variants’ function in EMT that would contribute to a better understanding of EMT itself and EMT-related diseases.

## 1. Epithelial-To-Mesenchymal Transition

Many key steps during embryogenesis result in the formation of new cell types with unique features. They become morphologically visible when individual cells or tissues are generated by cell delamination during a process called epithelial-to-mesenchymal transition (EMT) or by cell clustering and re-epithelialization during mesenchymal-to-epithelial transition (MET). EMT is categorized into three types, the developmental EMT (type I) is required for mesoderm formation, neural crest delamination, establishment of the heart valve, palatogenesis, and myogenesis [1]. After tissue damage, EMT becomes activated during fibrosis and wound healing (type II). EMT is also aberrantly activated during tumorigenesis when cancer cells start to disseminate, invade, and form metastases (type III) [2,3,4].

Cytoskeletal rearrangements and modulation of the expression of many different genes, including cell adhesion molecules, are the hallmarks of EMT and MET. The key event of a bona fide EMT is the downregulation of E-cadherin (E-cad) and the activation of N-cadherin (N-cad) expression that leads to a loss of cell polarity, adherent morphology, and of the epithelial gene signature [1,5]. In exchange, they acquire an unpolarized mesenchymal morphology combined with increased cell motility and a mesenchymal gene signature, observed by an increased fibronectin and vimentin expression [2]. The induction of EMT is also integrated by many different signals but shares the activation of intracellular EMT inducers like Snail, Slug, Twist, Zeb1, Zeb2, and others, which are at the core of a transcriptional regulatory network controlling the EMT program [1,3]. The EMT transcription factors seem to play a context-specific role during EMT [6,7].

MET is viewed as the reverse process of EMT and is a fundamental embryonic program as well [8,9]. Here, mesenchymal cells acquire epithelial characteristics, including the loss of N-cad and reactivation of E-cad expression [1,2,3]. In addition to orchestrating morphogenetic events during embryogenesis, the process of MET is also utilized by disseminating tumor cells and required for colonization and the formation of metastasis at distant sites [1,3,10].

## 2. The Epigenetic Landscape of EMT

EMT can be activated by several signaling pathways, including the transforming growth factor-β (TGFβ), NOTCH, and Wnt/β-catenin pathways. In these pathways, the ligand-dependent initiation of EMT triggers a cascade of cellular signaling molecules, which are involved in activating the EMT transcription factors, which interact closely with several chromatin-modifying enzymes and complexes, such as DNA methyltransferases, histone acetyltransferases/histone deacetylases, and histone methyltransferases/lysine demethylases, resulting in global changes in DNA methylation and several histone marks (including, but not limited to, H3K4me3, H3K4Ac, H3K27me3, and H3K9me3) to accommodate the required activation of mesenchymal genes, such as motility and invasion genes, and the suppression of epithelial genes such as CDH1 [11,12]. Of note, the available data on the epigenetic regulation of EMT are scarce, and it was only recently that the role of histone variants in EMT has been addressed and partially uncovered.

## 3. Histone Variants

DNA in eukaryotes is packaged into chromatin by small, lysine-rich architectural proteins, called histones. Two copies of each core histone H2A, H2B, H3, and H4 form the histone octamer, around which 146bp of DNA are wrapped 1.7 times to assemble the nucleosome core particle (NCP)—the fundamental subunit of chromatin [13,14]. The NCPs, connected with the linker DNA, form the 10-nm chromatin filament, which, upon binding of the linker histone H1, folds into higher chromatin structures [15,16].

In addition to core histones, synthesized primarily in the S phase and deposited at replication forks, each cell expresses histone variants. Histone variants are nonallelic isoforms of conventional histones and, in contrast to conventional histones, are usually expressed during all phases of the cell cycle [17,18]. Histone variants are encoded by separate genes, often synthesized constitutively at low levels and incorporated differently into chromatin. There are reported variants for all canonical histones (except H4), which vary from conventional counterparts from almost no amino acid differences to extremely divergent changes [19]. Even minor sequence changes play a crucial function(s) in cellular plasticity due to their ability to change the process’s dynamic nature [20].

The histone variants of H2A form the largest family of identified histone variants. So far, numerous variants of H2A have been identified, including H2A.Z-1, H2A.Z-2, H2A.X, H2A Barr body-deficient (H2A.Bbd; also known as H2A.B), macroH2A1.1, macroH2A1.2, and macroH2A2 [21,22,23,24]. Similarly, in human somatic cells, six variants of H3 have been identified. These are H3.3, histone H3-like centromeric protein A (CENP-A), H3.1T, H3.5, H3.X (also known as H3.Y.2), and H3.Y (also known as H3.Y.1) [21,25]. H2B also exhibits histone variants [26]. A detailed listing of all canonical histones and their variants can be accessed using HistoneDB 2.0, a database intended as a dedicated resource to explore histones and their variants [27]. A novel histone variant for H4 was recently reported; H4G was found to regulate the expression of ribosomal DNA (rDNA) in breast cancer cells [28].

The incorporation of histone variants confers novel structural and functional properties to the nucleosomes and modulates both the structural and functional landscapes of chromatin [29,30,31,32]. The notion of the structural heterogeneity on chromatin has evolved to contribute to the nucleosomal infrastructure [33]. Dedicated chaperones assist the specific deposition of histone variants in chromatin [34,35,36,37]. Cells efficiently use histone variants to control all main nuclear processes, including transcription, mitosis, and DNA repair, under normal and pathological conditions [21,29,38,39]. For example, H2A.Z nucleosomes flank the transcription start sites (TSS) of active genes and genes poised for activation [40,41]. This suggested that H2A.Z is involved in the control of transcription. However, recent experiments using conditional knockout models revealed, in contrast to the existing dogma, that H2A.Z is dispensable for both basal and activated transcription in postmitotic cells [42].

The data on the involvement of H3.3 in transcriptional regulation are also contradictory [43,44,45]. However, both H2A.Z and H3.3 histone variants are likely to play an essential role in cell division [44,45,46,47]. macroH2A, the most deviant histone variant from the H2A family, was found in a complex with PARP-1 [48], pointing that, in addition to its presumed role in transcriptional control, it may also be implicated, as stated for H2A.Z and H2A.X, in the repair of damaged DNA. H2A.Bbd (H2A.B) and H2A.L2, the two histone variants expressed mainly during spermatogenesis, change the chromatin landscape upon insertion, a process necessary for the proper proceeding of spermatogenesis [49,50,51]. CENP-A, the centromeres’ epigenetic marker, forms a nucleosome with very peculiar properties, essential for active kinetochore assembly [39,52].

## 4. The Implication of Histone Variants in EMT

Recently, evidence has been reported illustrating the impact of some histone variants on the EMT program. In the following section, we will recapitulate the latest data on the involvement of distinct histone variants in EMT.

### 4.1. H2A.Z and EMT

H2A.Z is an evolutionary conserved H2A histone variant, and it has a 60% amino acid identity with canonical H2A. It is encoded by two genes, H2afz (coding for H2A.Z-1) and H2afv (coding for H2A.Z-2), which products differ by only three amino acid residues [23,53,54].

H2A.Z is essential for mouse embryonic development and is expressed in the blastocyst [21]. Mice embryos deficient in H2A.Z die at the preimplantation stage of the blastocyst [55]. It has also been shown that the loss of H2A.Z in *Xenopus laevis* impaired the cell movement required for the formation of the mesoderm and neural crest [56]. Since the formation of the mesoderm is critically dependent on EMT, it is plausible that H2A.Z might be a chromatin regulator of EMT.

**Figure 1:**
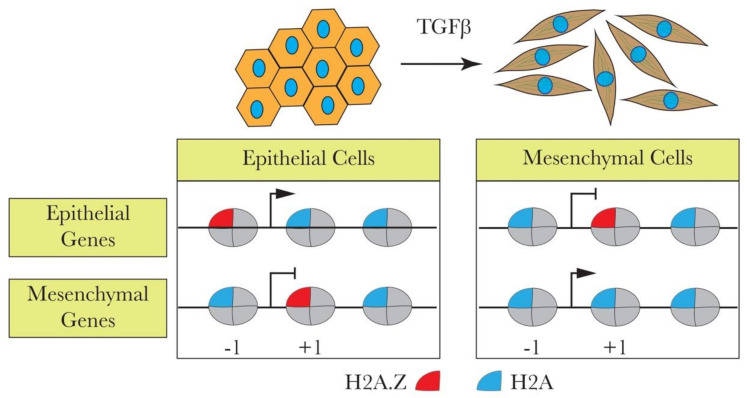
H2A.Z is associated with transforming growth factor beta (TGFβ)-induced epithelial-to-mesenchymal transition (EMT). TGFβ induces EMT by leading to H2A.Z loss from the epithelial and mesenchymal promoters, causing the up- or downregulation of the genes. The loss of H2A.Z from the -1 nucleosome in epithelial genes downregulates these genes. On the other hand, the loss of H2A.Z from +1 nucleosomes upon TGFβ induction upregulates mesenchymal genes.

Taking these data into consideration, Domaschenz et al. carried out a series of experiments to study the role of H2A.Z in EMT by using the Madin-Darby Canine Kidney (MDCK) cells as a model [57]. They showed that epithelial and mesenchymal gene promoters in these cells, as in many other cell types, contain H2A.Z nucleosomes in close proximity to their transcription start sites. Upon EMT induction by TGFβ treatment, a strong H2A.Z nucleosome reorganization took place, resulting in the loss of H2A.Z from both the epithelial and mesenchymal promoters [57]. The TGFβ-mediated loss of H2A.Z from the promoters of EMT/MET marker genes correlates with the gene expression changes. ChIP experiments revealed that the repression of epithelial genes correlated with the reduction of -1 and -2 H2A.Z promoter nucleosomes (the numbering of the nucleosomes is relative to the TSS). In contrast, the activation of mesenchymal genes was associated with the loss of the +1 H2A.Z nucleosome [57]. These data suggested that H2A.Z might be viewed as a Janus-type (“two faces”) player in EMT by serving as both an activator and repressor of epithelial and mesenchymal gene expression (see Figure 1). This conclusion was further supported in a series of experiments studying how the shRNA-mediated knockdown of H2A.Z affects MDCK cells. As expected, the loss of H2A.Z was associated with the acquisition of a higher migration rate, inability to form tight and structured colonies, and slower proliferation rate of the H2A.Z-depleted cells, the phenotypic features that are characteristic for mesenchymal cells. Notably, highly similar transcriptional profiles of EMT marker genes were observed for both H2A.Z knockdown and TGFβ-treated cells [57]. Taken together, these data revealed that the loss of H2A.Z mimics the TGFβ treatment and the acquisition of the mesenchymal phenotype in MDCK cells. It is intriguing how H2A.Z loss from the promoter regions of epithelial and mesenchymal genes leads to two different functional outcomes. However, H2A.Z appears to facilitate the access of permissive and repressive complexes to chromatin in embryonic stem cells during self-renewal and differentiation [58]. If such a mechanism also operates in somatic cells, it could be at the origin of the dual role that H2A.Z nucleosomes play during EMT.

In a recent paper, Nam and colleagues claimed that H2A.Z is associated with EMT in both SNU-449 and SK-Hep1 liver cancer cells [59]. Their data showed that H2A.Z is linked with cell migration and/or with cell migration signatures. Indeed, H2A.Z knockdown significantly suppressed the stimulated migratory and invasive responses and also affected the wound-healing ability of both cell types [59]. Besides, the absence of H2A.Z expression appeared to inhibit the expression of the mesenchymal marker fibronectin and activate the epithelial marker E-cadherin [60]. These data suggested that H2A.Z might be linked with the mesenchymal-to-epithelial transition. As for the relationship of H2A.Z with the migratory cell signatures, this effect could be attributed to the involvement of H2A.Z in cell proliferation. Taken as a whole, these studies indicate that H2A.Z, in tune with the reported data on its in vivo function in intestinal cells [61], is implicated in cellular homeostasis and plays various roles in different cellular contexts (Figure 1).

### 4.2. H2A.X is Implicated in EMT

The histone variant H2A.X is mainly associated with the DNA damage repair system induced by DNA double-strand breaks [62,63] and serves as a DNA damage sensor. It differs from H2A by a four-amino acid carboxy-terminal motif whose serine residue is the site for phosphorylation at sites of DNA double-stranded breaks. This phosphorylation is an early event in double-strand break repair and is thought to trigger the recruitment of the repair machinery [64].

**Figure 2:**
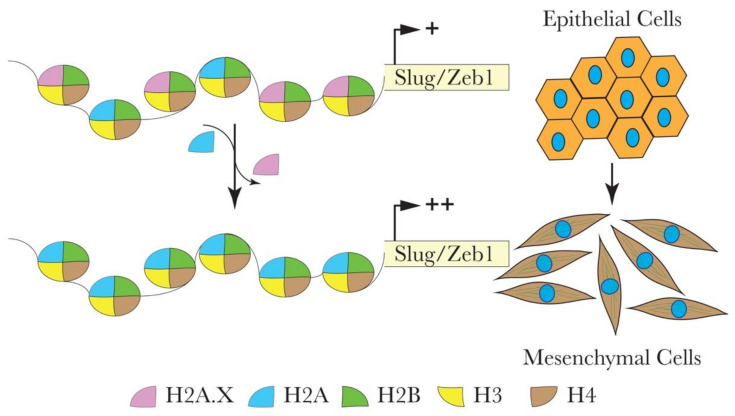
Hypothetical model for the role of H2A.X in the transcriptional regulation of Slug and ZEB1 during EMT in colon cancer HCT116 cells. H2A.X removal from the nucleosome leads to the enhanced enrichment of active chromatin marks within the promoters of Slug and ZEB1. This chromatin configuration enables the transcriptional activation of Slug and ZEB1. Elevated levels of Slug and ZEB1 are vital in mediating the expression of several EMT-related genes.

Deficiency of the histone variant H2A.X, which has an essential role in DNA repair and genome stability, activates EMT in some cancer types [65,66]. Weyemi et al. used a genome-wide differential gene expression analysis of H2A.X-deficient and control human colon cancer cells (HCT116) to show that H2A.X is involved in regulating EMT [65]. The loss of H2A.X induced mesenchymal-like characteristics, such as acquiring active chromatin transcription marks of the EMT transcription factors Slug and ZEB1, leading to their activation. Additionally, there was a significant correlation between H2A.X loss and the activation of a set of other mesenchymal genes (VIM, THBS1, VCAN, TGFB2, and ITGB4, among others) coupled with the repression of key epithelial genes (CDH1, RAB25, SERPINB5, and MAGI1). The data suggest that the transcription factors Slug and ZEB1 mediate the EMT driven by H2A.X loss as their co-silencing lead to the reversal of EMT in HCT116 cells (Figure 2). The reintroduction of H2A.X in knockout cells restored the expression of H2A.X to wildtype levels. The expected reversal of the phenotype was accompanied by the anticipated restoration of E-cad levels. Strikingly, the EMT marker genes remained elevated in the revertant cells, suggesting the establishment of a partial EMT state, which probably facilitated the metastatic colonization in xenograft models [65]. Partial EMT has been described recently by many research groups with subtle differences regarding the location and timing of their appearance during the acquisition of the metastatic state, yet they share the fact that these cells (the partial EMT cells) express E-cad along with mesenchymal markers, including the EMT inducers [67]. To the best of our knowledge, the precise mechanisms leading to the appearance of such a partial EMT state remain mostly unclear. The involvement of histone variants such as H2A.X in this phenotype is a step forward to better understanding partial EMT and extending a similar function to the other histone variants involved in EMT.

In another study, the authors used a human nontumorigenic breast cell line MCF10A and showed that the loss of H2A.X also results in a robust EMT activation [66]. Cells deficient for H2A.X exhibit enhanced migration and invasion, the activation of mesenchymal genes with a concomitant repression of epithelial genes. However, H2A.X plays a tissue-specific role by employing different transcription factors in different cellular contexts. For example, as described above in human colon cancer cells (HCT116), silencing of the histone variant H2A.X induced activation of the EMT transcription factors Slug and Zeb1 [65], while, as in the human nontumorigenic breast cell line MCF10A, it activates Twist1 and Slug [66].

### 4.3. Involvement of macroH2A in EMT/MET

**Figure 3:**
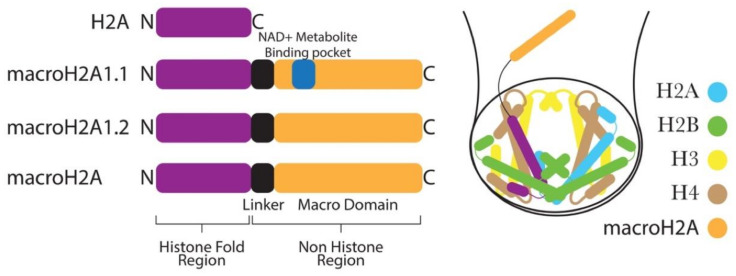
MacroH2A histone variants have a characteristic C-terminal nonhistone domain and a linker region. The nonhistone domain protrudes out from the nucleosome core.

Another histone variant that has been shown to play a role in EMT is macroH2A. It is the most deviant histone variant, with a unique tripartite structure composed of an amino-terminal H2A-like histone fold (amino acids 1–122), an unstructured and highly basic linker region (amino acids 132–160), and a globular macro domain (amino acids 161–370) that protrudes from the core nucleosome structure (Figure 3) [68,69]. The crystal structure of NCP containing the H2A-like domain of macroH2A is quite similar to canonical NCP, showing that the incorporation of the H2A-like domain of macroH2 into NCP causes minor rearrangements in the NCP [70,71,72].

In mammals, H2afy1 and H2afy2 genes encode two isoforms of macroH2A, termed macroH2A1 histone and macroH2A2 histone, respectively. The H2afy1 gene further produces two splice variants, known as macroH2A1.1 and macroH2A1.2, from the alternative splicing of mutually exclusive exons. These two splice variants differ in about the 30-amino acid region of the macrodomain [73,74]. Notably, the macrodomain of macroH2A1.1, but not that of macroH2A1.2, can bind the NAD+ metabolite O-acetyl-ADP-ribose (OAADPR) (Figure 3) [75]. In general, macroH2A-containing chromatin has been shown to be inhibitory to transcription in vivo [76]. Moreover, in vitro, macroH2A-containing nucleosomes are not accessible to transcription factors and are not remodeled by the chromatin remodeler SWI/SNF [77].

MacroH2A has been implicated in many biological processes, such as differentiation, somatic cell reprogramming, and cancer [21]. It also appears to function as an epigenetic stabilizer that establishes and maintains differentiated states and acts as a barrier to reprogramming [78]. Recently, Pliatska et al. showed that macroH2A1.2 blocks reprogramming and contributes to preserving the cell identity by trapping cells at the mesenchymal-to-epithelial transition state [79]. The lentiviral expression of OCT4, SOX2, KLF4, and c-MYC, together with the depletion of macroH2A1.2 in mesoderm-derived human fibroblasts (hFBSs), successfully reprogrammed the hFBSs into human iPSCs. Knocking down macroH2A1.2 seems to be critical here, as overexpression of this variant makes hFBSs resistant to reprogramming. Histone variant macroH2A1.2 knockdown in hFBSs showed a significantly reduced expression of critical mesenchymal genes SNAI1 and N-cad, together with the earlier shift of expression of the epithelial marker E-cad. These data suggest that reduced levels of macroH2A1.2 enhance cellular programming by facilitating the N-cad-to-E-cad transcriptional switch and, thus, MET.

In yet another study, macroH2A1 was shown to play an isoform-specific role in suppressing EMT [80]. Here, the authors observed a loss of macroH2A1 during EMT induced by overexpression of the transcription factors SNAI1 (Snail) or TWIST1 (Twist). This suggested that macroH2A1 plays a role in EMT, and it appears to be involved in blocking EMT induction. However, the ectopic expression of macroH2A1 isoforms in these cells prevented EMT induction in only the cells expressing macroH2A1.1 but not macroH2A1.2, suggesting an isoform-specific involvement in inhibiting EMT. This raises the question of whether endogenous macroH2A1.1 absence alone is sufficient to induce EMT. The CRISPR-mediated knockout of macroH2A isoforms in immortalized human mammary epithelial cells (HMLE) either individually or in combination showed that the knockout of neither one of the individual macroH2A1.1 variants was sufficient to induce EMT in HMLE cells [80]. These data clearly illustrate that the loss of macroH2A1.1 is insufficient to induce EMT but may be a barrier to reprogramming induced by other EMT inducers. The ability of macroH2A1.1 to suppress EMT induction is in tune with its function, which is the reduction of PARP-1 protein expression [74]. Indeed, PARP-1 is implicated in increasing the Snail level, a repressor of E-cadherin and a key player in EMT progression. This also explains why macroH2A1.2, which lacks the NAD+ metabolite-binding domain and is not involved in the control of PARP-1 expression, cannot suppress EMT.

## 5. Conclusions

Histone variants are used by the cells to shape and model the genome landscape at wish, which, in turn, controls major cellular events. This general function of histone variants is also exemplified in the case of EMT. Due to the limited number of studies implicating histone variants in EMT, it is challenging to anticipate histone variants’ involvement in varying EMT models induced by other signals. However, the role of histone variants in EMT might be independent of the inducing signal and the corresponding pathway, placing the function of such histone variants downstream of these signals and closer to the function of the EMT-inducing transcription factors. The depletion of either H2A.Z or H2A.X in epithelial cells resulted in the spontaneous induction of EMT, suggesting that these two histone variants are required for preserving the epithelial cell phenotype. However, other data reviewed here suggested that H2A.Z is also acting as a “gatekeeper” for the mesenchymal cell state. As for macroH2A, it is important for both EMT and MET, but its role is indirect and requires the concerted action of macroH2A with several transcription factors. In this review, the summarized roles of histone variants in EMT/MET identified histone variants as major actors required to preserve the specific cell phenotype and the cell homeostasis, in general.
